# 

**Published:** 1887-09-24

**Authors:** 


### The Hospital.

**An Institution, Family, and Parochial Journal of Hospitals, Asylums, and all Agencies for the Care of the Sick, Criticism and News.**

SATURDAY, SEPTEMBER 24TH, 1887.

### NOTICE TO ADVERTISERS.

THE scale of charges for small prepaid Advertisements—Situations Wanted, Situations Vacant, etc., etc., is as follows :—

| | s. | d. |
|---|---|---|
| One insertion of three lines | 1 | 0 |
| Three „ „ „ | 2 | 6 |
| Per line afterwards | 0 | 6 |

*A line averages eight words.*

All copy for Advertisements must be sent to A. P. WATT, 2, PATERNOSTER SQUARE, E.C., by first post on the Tuesday *preceding* publication.

### NOTICE TO CORRESPONDENTS.

All correspondence must be written on one side of the paper only.

We cannot undertake to return MSS. not used.

**Communications,** whether intended for insertion or for private information, must be authenticated by the names and addresses of the writers, not necessarily for publication.

**Local papers** containing reports or news paragraphs **should be marked.**

It is especially requested that early intelligence of **local events of interest,** or which it may be thought desirable to bring under notice, may be sent direct to the Editor.

Communications respecting editorial matters should be addressed to the Editor, Norfolk House, Norfolk Street, Strand, London, W.C.

### THE CHILDREN'S CORNER.

**Fourth Quarterly Prize Competition.**
Began 2nd July, 1887 ; ends 24th Sept., 1887.

PRIZES.

FOR MOST CORRECT ANSWERS TO PUZZLES.

| | | |
|---|---|---|
| 1st .. .. Thirty Shillings | 3rd .. .. Ten Shillings |
| 2nd .. .. Twenty „ | 4th .. .. Five „ |

FOR NEATEST ANSWERS TO PUZZLES.
Five Shillings.

FOR BEST LITTLE LETTERS.
Ten Shillings.

I must remind you all that this is the *last* week of the Fourth Competition. Lately, several of my correspondents have failed to send me answers to the Puzzles. This I know is owing to the "Holidays," which I hope they have all enjoyed, but now that lessons have begun again, I trust that they will not only return with new zest to the competition themselves, but will also each one induce one or more friends to join in the contest.

**Puzzles—Thirteenth Week.**

No. 50. ARITHMETICAL PUZZLE. A gentleman meeting a man driving sheep along a road, asked him : "Where are you driving that score of sheep ?" The man replied : "If I had as many more, and half as many more, and two sheep and a half, I should then have a score." How many sheep had he ?

No. 51. Convert the following into a sentence :

Captain B B B B sent his C C C C to the

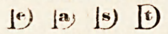

No. 52. GEOGRAPHICAL ACROSTIC. The initials give the name of a country, and the finals something for which it is celebrated. 1. A city in Somersetshire. 2. A month in the year. 3. One of the United States. 4. A kind of lizard. 5. A river in France which gives its name to four Departments.

No. 53. TRANSPOSITION. Transpose an English river, and it will bring an English town to view.

**Letters—Thirteenth Week.**

The letter-subject for next week will be "Blackberrying."

**Results of Eleventh Week—Puzzles.**

No. 42. EPITAPH PUZZLE.

Beneath in the dust, the mouldy old crust
Of Nell Bachellor lately was shown,
Who was skill'd in the arts
Of pies, custards, and tarts,
And knew every use of the oven,
When she'd liv'd long enough,
She made her last puff ;
Now here she doth lie,
In hopes that her crust will be rais'd.

No. 43. CHARADE. All guessed the great animal painter, Landseer.

No. 44. A small fishpond resembles a birdcage when it has a *perch* in it.

No. 45. DECAPITATION. Od(d), Co (o)=Cod.

*All right*—The Eda, Agatha, Mount Edgcumbe, Umslopogaas, Scrooge, Toby ; *Three right*—Fern, Skye, Ossian, A. G. S. McCulloch, Princess Ida, A. C. K. ; *Two right*—Socrates.

**Letters.**

I have received some very touching and interesting stories about *dogs* this week, showing their faithfulness and sagacity. Socrates sends me the following true and affecting story of the fidelity of a Newfoundland dog, which I am sure will move the hearts of all who read it :—

The early part of our childhood was spent in the country. Our family was only four in number, my parents, my sister Daisy, and myself. When she was four years old she had a beautiful Newfoundland dog sent to her by our uncle. It had such a noble face, and resembled a lion so much, that we named it Leo. It was greatly attached to Daisy, and would do anything she told him. She was playing on the banks of the river that flowed through our grounds one day, when she fell in, and would have been drowned had not our dog Leo bounded in, and brought her safely to land, thus endearing himself doubly to us. At the early age of six Daisy was attacked with the cholera, which was raging through the village, and the following week she died, and was buried in our family vault. During her illness Leo showed unmistakable signs of grief, and on the day of her death found his way into her room, and placing his nose on her cold forehead, uttered a howl of anguish, and on the day of her funeral disappeared. My mother never recovered from the loss of Daisy, and in six months afterwards was carried to the grave. When the men opened the vault to take in my mother, they saw stretched out on my sister's coffin the skeleton of a dog, with its head resting on the place where her face was. On going nearer they discovered by the large bones that it was Leo. As we could get no information, we concluded that he had followed his young mistress to the grave, and entered unperceived, and upon finding himself shut in, had smelt out the place of Daisy's coffin, and stretching himself upon it, had at last died of starvation, being even unto death "Faithful and True."

Skye writes :—

We had a fine dog in Skye, but we had to leave him behind. He followed us through the water, and jumped into the steamboat, and had to be carried on shore by force, but escaped, and swam after us for a considerable distance, until he saw he was making no head, then he sat on the shore howling piteously. We had told our old manservant to take charge of him. He was very kind to him, and I believe after he forgot us he lived happy till he died, and was blind of an eye.

Want of space prevents my remarking further upon other letters which I have received, some of which I hope to insert on another occasion.

Three marks each :—Socrates, Ossian, Skye, Princess Ida, Ellie. Two marks each :—Toby, Mount Edgcumbe.

Answers must be addressed to the "PUZZLE EDITOR," THE HOSPITAL, Norfolk House, Norfolk Street, Strand, W.C., not later than Thursday next. The "Children's Corner" Coupon (see advertisement pages) must be enclosed.

The address and full Christian name and surname, and the age of the competitor, must be stated in all replies. A *nom de plume* may be added for the purposes of publication.

### AMUSEMENTS WITH PROFIT.

### PRIZE COMPETITIONS.

**Quarterly Prize Competition.**
Began 23rd July ; ends 15th October.

**No. 19. Double Acrostic.**

Of race "unspeakable," a king
At whom all Europe takes a fling ;
My last his country's synonym,
Much liked by gourmands, Christmas whim.

I am a place, I am a blot,
I cannot change myself a jot.
An ablative of Latin word
For custom, oft in law courts heard.
A father whom ingratitude
To madness and to death pursued.
I was a ruffler's instrument
When once on murder he was bent.
A blow's an insult hard to bear,
Yet mine's an honour anywhere.
A lady's name or town am I,
Or saucy brig more frequently.

**No. 20. Convalescence.**

Credit will be given for the best short practical paper containing suggestions for the Amusement of Convalescents.

**Answers.**

No. 15. DOUBLE ACROSTIC.

| | | |
|---|---|---|
| T | histl | E |
| R | azo | R |
| U | ttoxete | R |
| T | oled | O |
| H | ugger-mugge | R |

Correct answers have been received from Brownie, Gretta, Condorcet, Mater, Perseverance, V. W.

No. 16. RHYTHMICAL RECREATION ON "HOME."

In response to the above, we have received many and various contributions. Most of our correspondents have defined "Home" in its simple sense, instead of taking the wider and more extended view of the good that may be done by making life cheerful to those who have to fight for themselves in the world. The following is one of the exceptions :

Blessed the home of love ! that man most truly blest,
With life's clouds lightened, life's griefs cheered by love serene ;
More from the outward strife he turneth, finding rest,
Amid life's cares preserving thus a cheerful mien ;
While in his happiness he encountereth one
Whose home is loveless, and distressed with bitter need ;
Forth goes he to his succour till the work be done,
To show by love to others what is love indeed.

Answers have been received from V. W., Ellice, Brownie, K. M., Aralc, Condorcet, Gretta, Mater, Perseverance.

Answers must be addressed to THE PRIZE EDITOR, AMUSEMENTS WITH PROFIT, THE HOSPITAL, Norfolk House, Norfolk Street, Strand, W.C., not later than the *first* post on Friday next. The full name and address must in all cases be given, but a *nom de plume* may be added for publication. Only one side of the paper should be written on. The "Amusements with Profit" Coupon (see advertisement pages) must be enclosed with replies.

### THE IN- AND OUT-PATIENTS' HOSPITAL DIARY.

This Diary is so arranged as to make some portion of it appear every week, and the whole in each monthly part. It has been very difficult to compile, and corrections will at all times be gratefully received. It has been suggested that similar information about the larger Provincial and Scotch Hospitals would be useful to many readers. We should be glad to hear if this is felt to be the case.

### CHILDREN'S DISEASES.—*continued.*

| Hospitals and Terms of Admission. | Physicians, Surgeons, and Medical Officers, with Days and Hours of Attendance. |
|---|---|
| Samaritan Free Hospital for Women and Children, Lower Seymour Street, W.; Branch, 1, Dorset St., W. Sec., Mr. G. Scudamore. In-patients must be between 2 and 12. Out-patients Department (free) is at Edward's Mews, Duke Street, W. | *In-Physician*, Dr. W. H. Day. Daily, 2 *In-Surgeon*, Mr. W. A. Meredith. Daily, 2 *Out-Physicians*, Drs. M. Prickett and Amand Routh, W. S. 1.30 *Out-Surgeons*, Messrs. W. A. Meredith and J. D. Malcolm, M. Th.; A. H. G. Doran and W. S. A. Griffith, Tu. F., 1.30 |
| Victoria Hosp. for Children, Queen's Road, Chelsea, S.W. Sec., Capt. W. C. Blount, R.N. Age of boys, 2 to 12 years; age of girls, 2 to 16. Admission for in-patients by subscriber's letter. Accidents and urgent cases free at all times | *In-Physicians*, Drs. J. Evans, M. Th. 2; Ridge-Jones, Tu. F. 2 *In-Surgeons*, Messrs. Pick, Tu. F. 9.15; Clutton, M. Th. 4.30 *Out-Physicians*, Drs. A. Venn, W. S. 9.30; C. Fox, M. Th. 1.30; F. D. Drewitt, Tu. F. 9; J. Philpot, M. Th. 9 *Out-Surgeons*, Messrs. F. Churchill, Tu. F. 10; W. Pye, M. Th. 9.30 |
| West London Hospital, Hammersmith rd, W. | Daily, 2. *See* General Hospitals |

### CONSUMPTION AND DISEASES OF THE CHEST.

| Hospitals and Terms of Admission. | Physicians, Surgeons, and Medical Officers, with Days and Hours of Attendance. |
|---|---|
| City of London Hospital for Diseases of the Chest, Victoria Park, E. Sec., Mr. T. Storrar Smith By subscriber's letter available for 6 weeks. | *In-Physicians*, Drs. J. Thorowgood, S.; E. Smith, M.; J. Fothergill, W.; S. West, F.; G. A. Heron, W.; V. D. Harris, Tu. Hour, 2 *Out-Physicians*, Drs. V. D. Harris, Tu.; J. A. Ormerod, Th.; E. C. Beale, Tu. F.; B. Fenwick, W. S.; A. Money, W. S.; L. E. Shaw, M. Th. Hour, 2 |
| Hospital for Consumption, Fulham road, Brompton, S.W. Sec., Mr. H. Dobbin Admission by letter of recommendation, but, where unobtainable, application may be made to the Secretary at the Hospital. | *In-Physicians*, Drs. E. S. Thompson, M. Th.; C. T. Williams, M. Th.; R. D. Powell, Tu. F.; J. Tatham, W. S.; R. E. Thompson, W. S.; F. T. Roberts, Tu. F. Hours, 2 to 4 *In-Surgeon*, Mr. R. J. Godlee, F. *Out-Physicians*, Drs. T. H. Green, J. M. Bruce, M. Th.; J. K. Fowler, W. S.; P. Kidd and C. Y. Biss, Tu. F.; T. D. Acland, W. S. Hour, 12 |
| North London Hospital for Consumption and Diseases of the Chest, Mount Vernon, Hampstead, N. Sec., Mr. L. F. Hill Admission by subscriber's letter available for 6 weeks. | *In-Physicians*, Drs. A. Evershed, Tu.; B. O'Connor, F.; D. Pidcock, F. Hour, 10.30 *Out-Physicians*, Drs. B. O'Connor, D. Pidcock, J. E. Squire. Daily, 2 (attend at Out-patients' Branch, 216, Tottenham Court Road, W.) |
| Royal Hospital for Diseases of the Chest, 231, City Rd., E.C. Sec., Mr. John J. Austin By Governor's letter, available for six weeks | *In-Physicians*, Drs. Hensley, W.; Gilbart-Smith, M.; Finlay, Th. *In-Surgeon*, Mr. A. Pearce-Gould, M. Tu. W. Th. F. 2, S. 9 *Out-Physicians*, Drs. White, Tu. F.; Pringle, M. Th.; O. Browne, W. S.; A. Davies, M. F.; H. Habershon, W. S.; E. Stewart, Tu. Th. Hour, 1; S. 9 |
| Royal National Hosp. for Consumption and Diseases of the Chest, Ventnor. Offices, 34, Craven St., Strand, W.C. Sec., Mr. E. Morgan Admits only incipient cases by Governor's letter. | *In-and Out-Physicians*, Drs. J. G. Sinclair Coghill, and R. Robertson attend daily (at Ventnor) on out-patients at 11; on in-patients at noon *In- and Out-Surgeons*, Messrs. Whitehead, Williamson |

### EAR CASES.

| Hospitals and Terms of Admission. | Physicians, Surgeons, and Medical Officers, with Days and Hours of Attendance. |
|---|---|
| Central London Throat and Ear Hosp., Gray's Inn Rd., W.C. Sec., Mr. R. Kershaw Free, but when able, a small payment is expected | *In- and Out-Surgeons*, Mr. Lennox Browne, M, Th. 2.30; Dr. Orwin, W. S. 2.30; Dr. Grant, Tu. 5, Th. 2.30; Messrs. P. S. Jakins, M. W. 2.30; F. 5; T. Carmalt Jones, M. Th. S. 2.30 |
| Charing Cross Hospital, West Strand, W.C. | *In- and Out-Surgeon*, Mr. Cantlie, F. 9 |
| Great Northern Central Hospital, Caledonian Rd, N. | *In- and Out-Surgeon*, Mr. A. E. Cumberbatch, W. 9 |
| Guy's Hospital, St. Thomas's Street, S.E. | *In- and Out-Surgeon*, Mr. Purves, Tu. F. 12.30 |
| Hospital for Diseases of the Throat, Golden Square, W. Sec., Mr. W. T. Sharp | *Physicians*, Drs. Wolfenden, Tu. F. 2.30; Macdonald, Tu. F. 6.30; Bond, W. S. 2.30 *Surgeon*, Mr. T. M. Hovell, M. Th. 2.30 |

### EAR CASES—*Continued.*

| Hospitals and Terms of Admission. | Physicians, Surgeons, and Medical Officers, with Days and Hours of Attendance. |
|---|---|
| King's College Hospital, Portugal Street, W.C. | *In- and Out- Surgeon*, Mr. Pritchard Th. 2.30 |
| London Hospital, Whitechapel Road, E. | *In- and Out-Surgeons*, Dr. Woakes, Mr. T. M. Hovell, S. 9 |
| Middlesex Hospital, Berners Street, W. Free. | *Out-Surgeon*, Mr. Hensman, Tu. 9 |
| National Hosp. for the Paralysed, etc., Queen Sq., W.C. | *In- and Out-Surgeon*, Mr. A. E. Cumberbatch, W. 2 |
| North West London Hospital, 18, Kentish Town Rd. | *Out-Surgeon*, Mr. Collier, M. 1.30 |
| St. Bartholomew's Hospital, Smithfield, E.C. | *Surgeon*, Mr. Cumberbatch, Tu. F. 2 |
| St. George's Hospital, Hyde Park Corner, S.W. | *In- and Out-Surgeon*, Sir W. B. Dalby, Tu. 1.30 |
| St. Mary's Hospital, Cambridge Place, Paddington, W. | *In- and Out-Surgeon*, Mr. G. P. Field, M. Thur. 3 |
| St. Thomas's Hospital, Westminster Bridge Road, S.E. | *Out-Surgeon*, Mr. Clutton, M. 1.30 |
| University College Hospital, Gower Street, W.C. | *In- and Out-Surgeon*, Mr. Barker, Th. 9 |
| Westminster Hospital, Broad Sanctuary, S.W. | *In- and Out-Surgeon*, Mr. Barrow, Tu. F. 9 |

### EYE DISEASES.

| Hospitals and Terms of Admission. | Physicians, Surgeons, and Medical Officers, with Days and Hours of Attendance. |
|---|---|
| Central London Ophthalmic Hosp., 238a, Gray's Inn Rd., W.C. Sec., Mr. G. H. Leah Admission free | *Surgeons*, Messrs. T. Archer, G. Abbott, W. Charnley, W. Jessop, E. Clarke, J. R. Kemp and Granville Barker (House Surgeon). Daily, 1 |
| Evelina Hosp. for Sick Children, Southwark Bridge Rd. | *Surgeon*, Dr. W. Brailey, Th. 1 |
| Great Northern Central Hospital, Caledonian Rd., N. | *Surgeon*, Mr. Hutchinson, Jnr., Tu. F. 9.30 |
| Guy's Hospital, St. Thomas's Street, S.E. | *Surgeons*, Mr. Higgens, Dr. Brailey, Tu. F. 12.30 |
| Hospital for Sick Children, 49, Gt. Ormond Street, W.C. | *Surgeon*, Mr. R. Marcus Gunn, Tu. 2 |
| King's College Hospital, Portugal Street, W.C. | *Surgeon*, Mr. McHardy, M. W. Th. 1.30 |
| London Hospital, Whitechapel Road, E. | *Surgeons*, Messrs. Waren Tay, S. 9; Eve, Tu. 9 |
| Middlesex Hospital, Berners Street, W. | *Surgeon*, Mr. Lang, Tu. F. 9 |
| National Hosp. for the Paralysed, etc. Queen Sq., W.C. | *Surgeons*, Messrs. R. Brudenell Carter F. 4; R. Marcus Gunn, Tu. 4 |
| North-West London Hosp. 18, Kentish Town Rd., N.W. | *Surgeon*, Dr. W. Collins, F. 3.30 |
| Paddington Green Children's Hosp., Paddington Grn. | *Surgeon*, Mr. W. H. H. Jessop, Th. 9 |
| Royal Free Hospital, Gray's Inn Road, W.C. | *Surgeon*, Mr. Mackinlay, M. F. 9 |
| Royal London Ophthalmic Hospital, Blomfield Street, Moorfields, E.C. Sec., Mr. R. J. Newstead Admission free to the poor. | *Surgeons*, Messrs. J. W. Hulke, W. S.; G. Lawson, Tu. F.; J. Couper, Tu. F.; W. Tay, M. Th.; J. Tweedy, Tu. F.; E. Nettleship, W. S.; R. M. Gunn, W. S.; Lang, M. Th. Hours, 8 to 10 |
| Royal South London Ophthalmic Hosp., 6, St. George's Circus, S.E. Sec., Mr. C. Comyn Free to the necessitous poor; or by payment | *Surgeons*, Messrs. Watson, McHardy. Daily, 2 |
| Royal Westminster Ophthalmic Hospital, King William Street, W.C. Sec., Mr. T. Beattie-Campbell Poor and accidents free | *In- and Out-Surgeons*, Messrs. Power, M. F.; Frost, M. Th.; Rouse, Tu. S.; Hartridge, Tu. F., Macnamara, M. Th.; Cowell, W. S.; Juler, W. S. Hour, 1 |
| St. Bartholomew's Hospital, Smithfield, E.C. | *Surgeons*, Messrs. Power, Th. 2; Vernon, Tu. S. 2.30 |
| St. George's Hospital, Hyde Park Corner, S.W. | *Surgeons*, Messrs. B. Carter, Frost, M. Th. 2; F. 1.15; W. S. 2 |
| St. Mary's Hospital, Cambridge Place, Paddington. W. | *Surgeons*, Messrs. A. Critchett, H. Juler, Tu. F. S. 9 |
| St. Thomas's Hospital, Westminster Bridge Road, S.E. | *Surgeons*, Messrs. Nettleship, Tu. Th. F. 1.30; Lawford, M. W. 1.30 |
| University College Hospital, Gower Street, W.C. | *Surgeon*, Mr. Tweedy, M. Tu. Th. F. 2 |
| West London Hospital, Hammersmith Road, W. | *Surgeons*, Messrs. B. Vernon, H. Dunn, Tu. Th. S. 2 |
| Westminster Hospital, Broad Sanctuary, S.W. | *Surgeon*, Mr. Cowell, M. Th. 2 |
| Western Ophthalmic Hosp. 153 and 155, Marylebone Rd., W. Sec., Mr. G. E. Manton. By letter or small payments; free to poor | *Surgeons*, Drs. Miller, M.; Collins, F.; Wheatly, M. Th.; Messrs. Carmal, Jones, W.; Buxton, Tu. F.; Nunn, W. S. Hour, 2 |

